# Comparative Cranial and Postcranial Osteology of *Blanus* Species (Squamata: Amphisbaenia) from Türkiye: Insights from Morphological Evolution and Phylogeny

**DOI:** 10.3390/life15081263

**Published:** 2025-08-09

**Authors:** Elif Yıldırım Caynak, Kamil Candan, Yusuf Kumlutaş, Çetin Ilgaz, Ahmet Gökay Korkmaz, Emine Beyza Yazar, Eda Şen, Ecem Büşra Hastürk, Sezen Birlik, Esra Akat Çömden, Serkan Gül

**Affiliations:** 1 Department of Biology, Faculty of Science, Dokuz Eylül University, 35390 İzmir, Türkiye; yildirim.elif@deu.edu.tr (E.Y.C.); kamil.candan@deu.edu.tr (K.C.); yusuf.kumlutas@deu.edu.tr (Y.K.); cetin.ilgaz@deu.edu.tr (Ç.I.); ahmetgokay.korkmaz@deu.edu.tr (A.G.K.); 2 Fauna and Flora Research and Application Center, Dokuz Eylül University, 35390 İzmir, Türkiye; 3 Departmant of Biology, Graduate School of Natural and Applied Sciences, Dokuz Eylül University, 35390 İzmir, Türkiye; yazar.eminebeyza@ogr.deu.edu.tr (E.B.Y.); eda.sen@ogr.deu.edu.tr (E.Ş.); hasturk.ecembusra@ogr.deu.edu.tr (E.B.H.); 4 The Ministry of Agriculture and Forestry Food Control Laboratory, Bornova, 35100 İzmir, Türkiye; sezen@uludag.edu.tr; 5 Department of Biology, Faculty of Science, Ege University, 35100 İzmir, Türkiye; esra.akat@ege.edu.tr; 6 Department of Biology, Faculty of Arts and Sciences, Recep Tayyip Erdogan University, 53100 Rize, Türkiye

**Keywords:** Blanidae, skeletal osteology, morphometrics, phylogeny

## Abstract

The genus *Blanus* (Amphisbaenia: Blanidae) comprises fossorial, limbless reptiles with cryptic external morphology, making species delimitation particularly challenging. This study presents a comprehensive comparative osteological and geometric morphometric investigation of three *Blanus* species distributed in Türkiye—*B. alexandri*, *B. aporus*, and *B. strauchi*. Using both dry and cleared-and-stained specimens, diagnostic variations in cranial and postcranial skeletal elements, especially elements within the nasal, maxilla, vomer, squamosal, dentary, and coronoid bones, as well as vertebral counts were identified. A geometric morphometric analysis of the dorsal and ventral cranial morphology revealed distinct shape differences, particularly separating *B. alexandri* from the other two species along principal component axes. A phylogenetic analysis based on 45 discrete osteological characters supported the monophyly of the eastern *Blanus* clade, with *B. alexandri* forming a distinct lineage from *B. aporus* and *B. strauchi*. These findings emphasize the significance of skeletal morphology for resolving phylogenetic relationships and highlight the role of osteological characters in refining species boundaries within cryptic reptilian taxa. The integrative approach employed here underscores the evolutionary distinctiveness of Anatolian *Blanus* and enhances our understanding of morphological evolution in amphisbaenians.

## 1. Introduction

The genus *Blanus* Wagler, 1830 (Amphisbaenia: Blanidae) includes seven limbless, fossorial species with a broad geographical distribution [1,2,3]. Three of these species are widely distributed in Türkiye [4]: *Blanus strauchi* [5] occurs in Western Anatolia and the southern regions of the country, extending as far as Antalya. *Blanus aporus* [6] ranges from Antalya to the vicinity of Adana. In contrast, *B. alexandri* [2] is found in the Eastern Mediterranean and Southeastern Anatolia.

Taxonomic studies of this genus have traditionally relied on morphological characteristics. However, the inability to distinguish species solely based on morphology—along with relatively minor morphological variation despite marked genetic divergence—has led to significant challenges for species delimitation. In this context, osteological analyses offer a valuable tool for resolving taxonomic ambiguities. Previous studies on amphisbaenians have primarily addressed phylogenetic relationships, osteology, and external morphology [1,2,7,8]. In addition, geometric morphometric approaches have been widely employed to explore shape variation across taxa [2,9,10,11]. Despite these efforts, osteological studies of amphisbaenians remain scarce (see Appendix A). Research on *Blanus* species has generally focused on aspects such as phylogeny, distribution, microhabitat preference, new locality records, activity patterns, ear morphology, and helminth fauna [8,12,13,14,15,16,17,18,19,20,21]. Osteological investigations of *Blanus* species date back to the 19th century [5,22,23,24,25,26,27], with the most comprehensive recent contribution provided by Villa et al. [28], who examined the cranial osteology of all extant species within the Blanidae family. Molecular studies have resolved as two major clades within the genus: eastern and western [3,8]. The eastern clade, which includes *B. alexandri*, *B. aporus*, and *B. strauchi*, all distributed in Türkiye, was the focus of detailed cranial osteological comparisons in Villa et al.’s study [28]. However, data on the appendicular skeleton of amphisbaenians remain limited [26,27,29]. Osteological studies conducted on blanid species are not limited solely to extant taxa but also encompass extinct representatives. These investigations particularly focus on the analysis of cranial elements (such as skull bones and the dentary) and the postcranial skeleton (notably vertebrae). Research addressing the morphology of fossil blanids has made significant contributions to elucidating the evolutionary relationships between both living and extinct species. Comparative studies of cranial structures and vertebrae clearly highlight the value of fossil-based osteological data in clarifying taxonomic and phylogenetic relationships within the Blanidae family [30,31,32,33,34].

In the present study, potential synapomorphies and diagnostic osteological traits for clades within Amphisbaenia were determined through parsimony optimization of skeletal characters. Comparative analysis of cranial and postcranial skeletal elements in three *Blanus* species (*B. alexandri*, *B. aporus*, and *B. strauchi*) revealed cranial differences when compared with the findings of Villa et al. [28]. The specific objectives of this study are: (1) to provide a detailed osteological description of skeletal elements; (2) to infer interspecific evolutionary relationships based on osteological data; and (3) to assess cranial shape variation (dorsal and ventral views) among the three species using geometric morphometric techniques.

## 2. Materials and Methods

### 2.1. Sampling

The specimens examined in this study are the collection materials stored at the Fauna and Flora Research and Application Center of Dokuz Eylül University (Table 1). Sex determination was not performed based on presence or absence of ovaries (in females) or hemipenes (in males), nor by cloacal structure.

### 2.2. Osteological Study

Osteological analyses were conducted on eight specimens per species. The cleared and double staining was performed according to the methodology of Wassersug [35]. Osteological terminology in the descriptions primarily follows Gans and Montero [36] and Villa et al. [28]. All anatomical descriptions and illustrations were prepared using a Leica DFC295 stereo microscope (Heerbrugg, Switzerland). The final illustrations were arranged using PAINT 3D (Microsoft 6.2410.13017.0). All osteological characters are shared in Appendix B.

### 2.3. Geometric Morphometric Analysis

**Figure 1:**
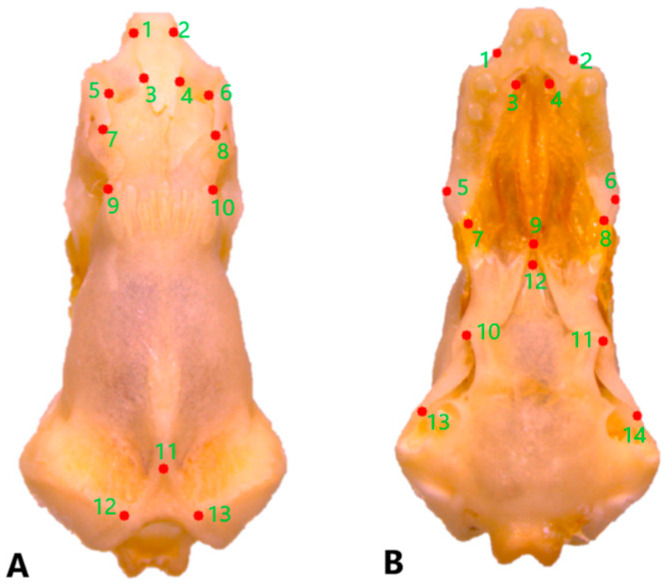
Position of the landmarks (**A**) on the dorsal and (**B**) on the ventral surfaces of a skull of *Blanus alexandri*.

For geometric morphometric analyses of dorsal cranial morphology, eight specimens were included for each *Blanus* species. Due to deformation in the palatal region, fewer specimens were available for the ventral cranial analysis: five for *B. alexandri*, six for *B. aporus*, and four for *B. strauchi*. Dorsal and ventral views of the skulls were photographed using a Leica DFC295 stereo microscope. Cranial landmarks were selected to represent biologically significant features, such as bone contacts and process tips. A total of 13 landmarks were digitized on the dorsal surface of the cranium and 14 on the ventral surface using tpsDig (v.2.16; Rohlf [37]) (Figure 1). Geometric shape information was extracted from the landmark coordinates by subjecting the landmark data to a generalized Procrustes analysis. The resulting Procrustes coordinates capture the symmetric component of variation after scaling, translating, and rotating all specimens to a common consensus and were used as shape variables in all subsequent analyses. Skull shape variation among *Blanus* species was assessed using principal component analysis (PCA) implemented in MorphoJ v. 1.05d.

### 2.4. Phylogenetic Reconstruction

Selected cranial and postcranial features of the adult skeleton were recorded in a data matrix comprising 14 taxa and 45 discrete characters with their corresponding states (Appendix C). Phylogenetic relationships were inferred using maximum parsimony analyses implemented in TNT version 1.5 [38,39] (version last modified 24 April 2025). Before initiating the analyses, memory allocation was adjusted to accommodate 10,000 trees. The analyses began with a ‘New Technology’ search strategy, employing the default settings of sectorial search, ratchet, drift, and tree fusing. The number of initial addition sequences was increased to 100 to ensure thorough sampling of tree space. Upon completion of this phase, a ‘Traditional Search’ was conducted using tree bisection and reconnection (TBR) branch swapping. This second phase utilized the trees retained in RAM from the previous search. To evaluate nodal support, decay index (Bremer support) values were calculated. This was achieved via TBR from the existing trees, allowing for the retention of suboptimal trees with up to 20 additional steps.

## 3. Results

### 3.1. Comparative Cranial Osteology

Villa et al. (2019) [28] conducted a detailed study on the cranial osteology of *Blanus* species. However, our study revealed differences in certain cranial bones compared with their findings. The observed discrepancies are summarized below.

#### 3.1.1. Nasal

**Figure 2:**
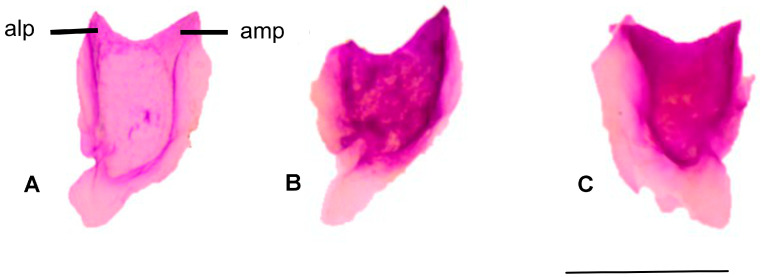
Dorsal view of the nasal bone. (**A**). *Blanus alexandri*, (**B**). *Blanus aporus*, and (**C**). *Blanus strauchi*. alp, anterolateral process; amp, anteromedial process. Scale bar = 2 mm.

In *Blanus aporus*, the anteromedial process of the nasal bone is longer than the anterolateral process, whereas in *B. alexandri* and *B. strauchi*, the two processes are nearly equal in length (Figure 2).

#### 3.1.2. Maxilla

**Figure 3:**
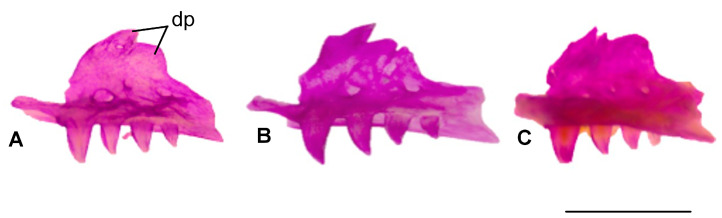
Dorsal view of the maxilla bone. (**A**). *Blanus alexandri*, (**B**). *Blanus aporus*, and (**C**). *Blanus strauchi*. dp, dorsal projections of the facial process. Scale bar = 2 mm.

The facial process shows species-specific projections in the dorsal end of the process. *B. alexandri* has two projections, while *B. aporus* and *B. strauchi* exhibit three. All three species typically possess four maxillary teeth that gradually decrease in size posteriorly (Figure 3).

#### 3.1.3. Parietal

**Figure 4:**
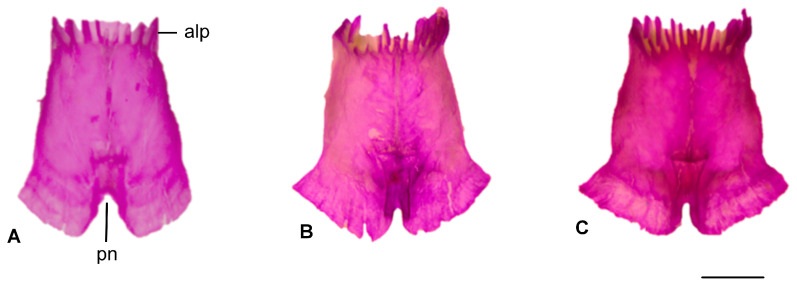
Parietal bone in dorsal view: (**A**). *Blanus alexandri*, (**B**). *Blanus aporus*, and (**C**). *Blanus strauchi*. alp, anterolateral process; pn, parietal notch. Scale bar = 2 mm.

A pair of parietal plates are detected, separated by a parietal notch at the posterior edge of the bone. This notch is wider and rounder in *Blanus alexandri*, whereas it is narrower and rounder in the other two blanid species (Figure 4).

#### 3.1.4. Prefrontal

**Figure 5:**
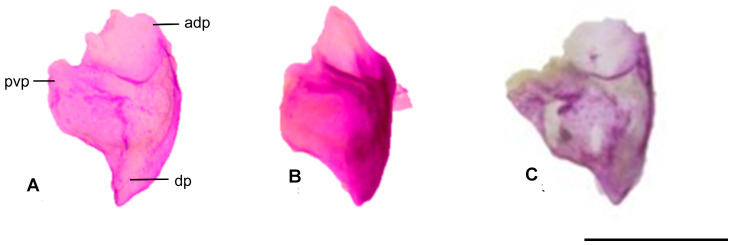
Prefrontal bone in dorsal view: (**A**). *Blanus alexandri*, (**B**). *Blanus aporus*, and (**C**)*. Blanus strauchi*. adp, anterodorsal process; dp, dorsal process, pvp, posteroventral process. Scale bar = 2 mm.

The prefrontal bone is one of the small, paired bones located anterolaterally in the skull. The anterodorsal process is situated at the dorsal boundary of the orbitonasal flange, while the posteroventral process is observed on the ventrolateral side. The dorsal process, which tapers to a point, is located on the posterodorsal side. In *Blanus alexandri*, the dorsal edge of the prefrontal bone is more concave, whereas in other species, it is flatter. In *Blanus aporus*, the posteroventral process is less developed (Figure 5).

#### 3.1.5. Squamosal

**Figure 6:**
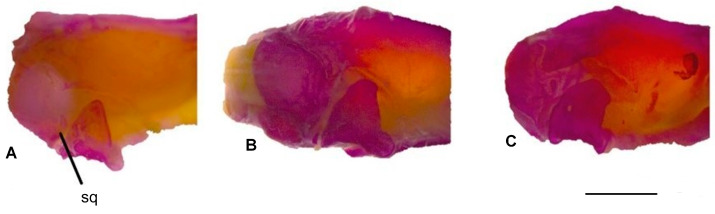
Squamosal bone in lateral view of the skull: (**A**). *Blanus alexandri*, (**B**). *Blanus aporus*, and (**C**). *Blanus strauchi*. sq, squamosal. Scale bar = 2 mm.

The squamosal bone appears as a thin, short, and reduced structure. It is present in all three *Blanus* species. In *B. strauchi* and *B. aporus*, the squamosal bone is highly reduced (Figure 4). However, in *B. alexandri*, the bone is approximately twice as long as in the other two species (Figure 6).

#### 3.1.6. Vomer

**Figure 7:**
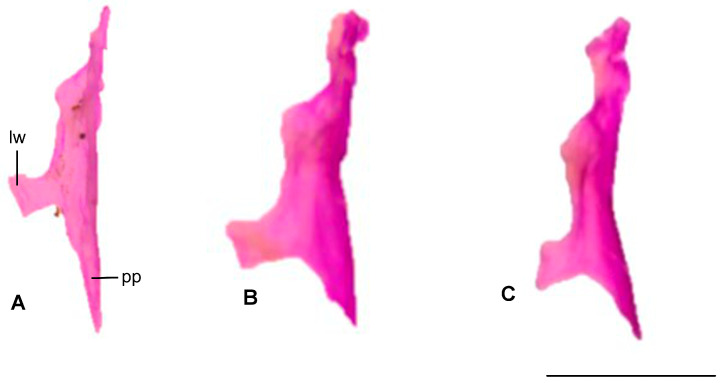
Vomer bone in the ventral view. (**A**). *Blanus alexandri*, (**B**). *Blanus aporus*, and (**C**). *Blanus strauchi*. lw, lateral wing; pp, posterior process. Scale bar = 2 mm.

The posterior process of *Blanus alexandri* is nearly twice as long as those of *B. aporus* and *B. strauchi*. The lateral wing is well developed and oriented anteriorly in *B. alexandri* and *B. aporus*, whereas it is oriented posteriorly in *B. strauchi* (Figure 7).

#### 3.1.7. Dentary

**Figure 8:**
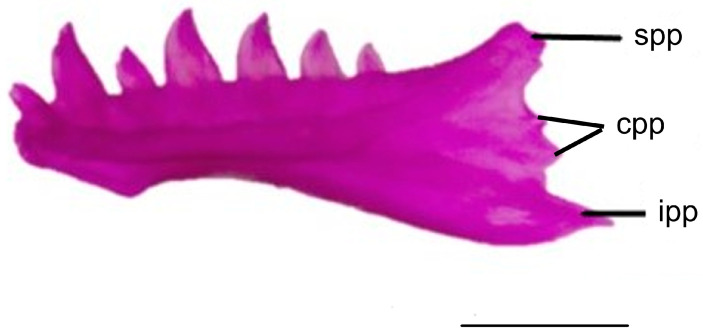
Dentary bone in lingual view of *Blanus aporus*. cpp, central posterior process; ipp, inferior posterior process; spp, superior posterior process. Scale bar = 2 mm.

The posterior end of the dental bone contains three processes. The superior posterior process extends posterodorsally and wraps laterally around the coronoid process. This process exhibits a wide and flat termination in *Blanus aporus* and *B. strauchi*, while in *B. alexandri*, it is long and tapers to a point. The inferior posterior process protrudes posteriorly, covering the ventrolateral side of the compound bone. The inferior posterior process is long and tapers in *B. alexandri*, *B. strauchi*, and *B. aporus*. The central posterior process is located between the inferior and superior posterior processes. In *B. alexandri*, the central posterior process is short and rounded, while in *B. strauchi*, it is also short but tapers to a point. In *B. aporus*, unlike the other two blanid species, the central posterior process is present in two instances (Figure 8).

#### 3.1.8. Coronoid

**Figure 9:**
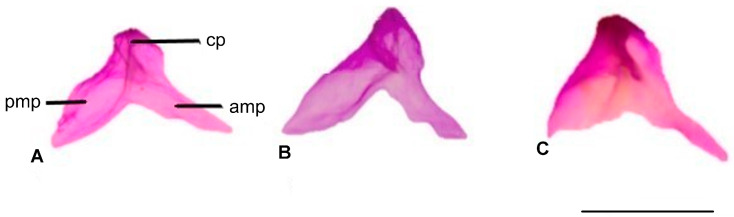
Coronoid bone in the lingual view. (**A**). *Blanus alexandri*, (**B**). *Blanus aporus*, and (**C**). *Blanus strauchi*. amp, anteromedial process; cp, coronoid process; pmp, posteromedial process. Scale bar = 2 mm.

The coronoid bone features three prominences: the anteromedial, posteromedial, and coronoid processes. The anteromedial process is narrow and pointed, while the posteromedial process is broad, tapering to a point in *Blanus strauchi* and *B. aporus* and rounded in *B. alexandri*. The anteromedial and posteromedial processes are separated at a wider angle along the median line in *B. strauchi* (Figure 9).

### 3.2. Osteological Characters Exhibiting Variation Among Blanus Species and Related Taxa

Several osteological features display considerable variation among *Blanus* species and closely related amphisbaenian taxa. These characters provide critical insights into the morphological diversity and evolutionary adaptations of these reptiles (see Appendix B).

### 3.3. General Postcranial Shape of Blanus Species

#### 3.3.1. The Vertebral Column

The vertebral column is composed of four distinct regions: cervical, thoracic, lumbar, and caudal. The number of vertebrae in each region is given in Table 2. The vertebral column is primarily distinguished by the presence or absence of ribs and the characteristics of the transverse processes. Cervical vertebrae are ribless, while thoracic vertebrae are rib-bearing. Although thoracic vertebrae are longer than those in the lumbar region, they are uniquely characterized by ribs that attach to each vertebra. In contrast, caudal vertebrae lack ribs but possess short transverse processes.

#### 3.3.2. Pelvic and Pectoral Girdles

**Figure 10:**
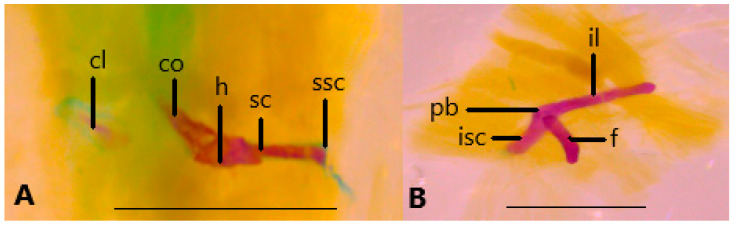
(**A**). Pectoral girdle in *Blanus alexandri* and (**B**). Pelvic girdle in *Blanus aporus*. cl, clavicula; co, coracoid; f, femur; h, humerus; il, ilium; isc, ischium; pb, pubis; sc, scapula; ssc, suprascapula. Scale bar = 2 mm.

The pectoral girdle of *Blanus* species consists of a small clavicular element and a rod-like coracoid, scapula, and suprascapula. In these species, none of the forelimb elements are present, except for a reduced humerus. All components of the pelvic girdle are present in *Blanus* species, although the pubis and ischium are both reduced in size and less developed than ilium. In the hindlimbs, only the femur is present, though it is reduced and slender in form (Figure 10).

### 3.4. Geometric Morphometric Analyses

#### 3.4.1. Dorsal Cranial Morphometrics

The PCA analysis indicated three main axes of group differentiation: PC1, PC2, and PC3 and explained, respectively, 34.64%, 18.56%, and 10.99% of the variation. The first three components account for 64.20% of the total variance. This indicates that a significant portion of the data can be effectively represented by these components, suggesting that a substantial amount of the underlying structure of the data is captured within them.

**Figure 11:**
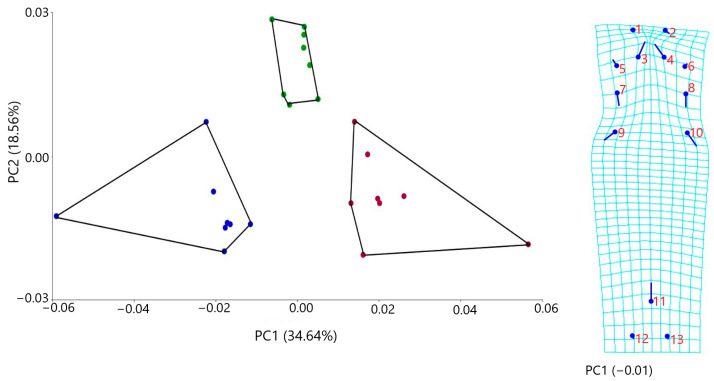
Shape changes in dorsal skulls among three blanids using a principal component analysis (blue dots, *B. alexandri*; red dots, *B. aporus*; green dots, *B. strauchi*).

Geometric morphometric analyses of dorsal cranial osteological features demonstrated clear morphological differentiation among the three *Blanus* species. Principal component 1 (PC1) effectively separated *Blanus alexandri* from *B. aporus* and *B. strauchi*, with the former exhibiting distinctly negative scores along this axis. The majority of shape variation was localized in the anterior region of the dorsal skull (Figure 11). Specifically, *B. alexandri* was characterized by a comparatively elongated and more medially oriented anteromedial process of the nasal bone, a longer and posteriorly directed facial process of the maxilla, a more extended and posterolaterally oriented dorsal process of the prefrontal, and a markedly deeper parietal notch (Figure 11). These osteological distinctions support the morphological separation of *B. alexandri* from its congeners and provide additional evidence for its species-level differentiation.

#### 3.4.2. Ventral Cranial Morphometrics

**Figure 12:**
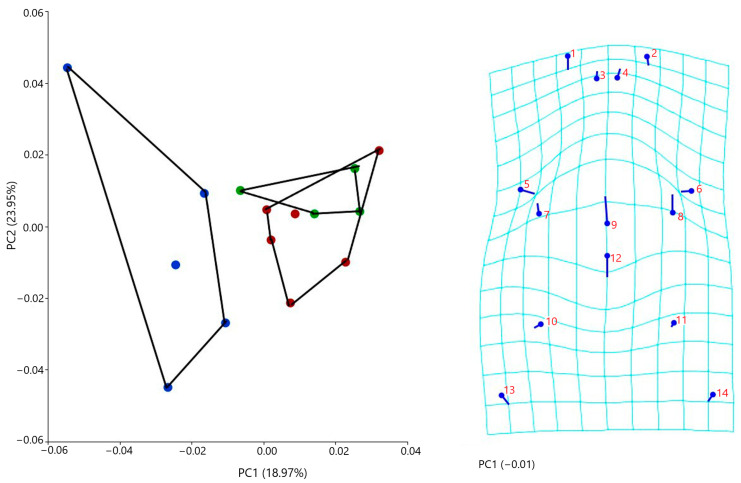
Shape changes in ventral skulls among three blanids using a principal component analysis (blue dots, *B. alexandri*; red dots, *B. aporus*; green dots, *B. strauchi*).

The PCA analysis revealed four principal axes of group differentiation, with the first four components explaining 70.14% of the total variance. Geometric morphometric analyses based on osteological features of the ventral skull demonstrated clear morphological differences among the three species. Along PC1, *Blanus alexandri* exhibited negative scores in comparison with the other two species (*B. aporus* and *B. strauchi*). Notably, the parasphenoid rostrum was shorter and more posteriorly oriented, while the posterior process of the vomer was shorter and more anteriorly directed (Figure 12). In addition, the posterior process of the maxilla was shorter and anteriorly oriented, and both the posterior process of the maxilla and the articulation between the ectopterygoid and maxilla were more medially directed (Figure 12).

### 3.5. Parsimony Reconstruction

**Figure 13:**
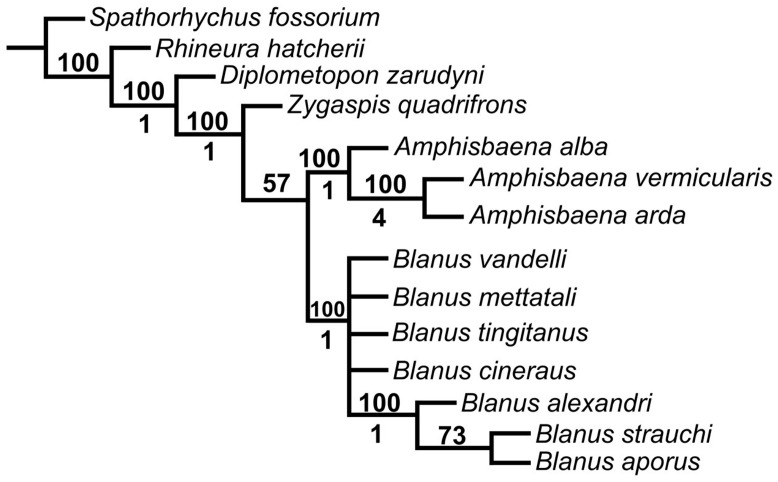
Majority-rule consensus tree from the parsimony analysis. Numbers above the nodes indicate the percentage of most parsimonious trees supporting each node. Numbers below the nodes represent Bremer support (decay index) values.

In the initial analysis, the ‘New Technology’ search reconstructed 32 most parsimonious trees (MPTs) with a best score of 114. The ‘Traditional search’, using the trees from RAM, inferred 10,000 MPTs (maximum memory). The majority-rule consensus tree provided better-resolved taxonomic implications; however, relationships among all *Blanus* species, except for the Turkish clade, were ambiguously reconstructed, resulting in a polytomy. Within the monophyletic study group, *B. strauchi* and *B. aporus* formed a sister clade, whereas *B. alexandri* branched as a more distantly related lineage (Figure 13).

## 4. Discussion

This study presents a comprehensive comparative osteological analysis of three *Blanus* species—*B. alexandri*, *B. aporus*, and *B. strauchi*—highlighting key differences in both cranial and postcranial skeletal elements. Morphological variations, particularly in the nasal, maxilla, squamosal, vomer, parietal, prefrontal, dentary, and coronoid bones, offer valuable insights into the evolutionary history and constraints of the genus. These osteological distinctions help address taxonomic challenges by supplementing traditionally limited morphological data with more diagnostic skeletal characteristics, thereby bridging the gap between morphological and molecular differentiation.

Our cranial osteological findings differ from those reported by Villa et al. [28] in several important aspects. Notable differences in the nasal, maxilla, squamosal, and vomer bones among *Blanus* species emphasize the importance of osteological features in distinguishing closely related taxa. For instance, in *B. aporus*, the anteromedial process of the nasal bone is distinctly longer than the anterolateral process, unlike in *B. alexandri* and *B. strauchi*, where both processes are nearly equal in length. Similarly, the variation in the number of projections from the maxillary facial process further underscores the species-specific distinctions observed in the cranial structure. These differences suggest that despite the overall morphological similarity among *Blanus* species, subtle osteological variations play a significant role in differentiating these species. Further diagnostic differences observed include the number of projections from the dorsal end of the facial process of the maxilla, the shape of the ascending nasal process of the premaxilla, the number of labial foramina on the maxilla, the number of finger-like projections on the posterior margin of each frontal bone, the number of finger-like projections on the anterior margin of the parietal bone, and the sagittal crest of the parietal bone. These osteological characters, in combination with clear biogeographic distributions [4], reinforce the evolutionary distinctiveness of the studied species and support Anatolia’s role as a biodiversity hotspot.

Although approximately 184 species are currently recognized within the Amphisbaenidae family [40], studies focused on cranial osteology remain scarce (Table 1). This study provides comparative cranial data from three *Blanus* species and seven amphisbaenian species (*Zygaspis quadrifrons* [41]; *Amphisbaena alba* [42]; *Diplometopon zarudnyi* [43]; *Rhineura hatcherii* [44]; *Amphisbaena arda* and *A. vermicularis* [45]; and *Spathorhychus fossorium* [46]). Our findings reveal differences in the nasal, frontal, parietal, premaxillary, and maxillary bones across these taxa. Species with a posterior process of the nasal bone approximately equal in length to the distal end of the ascending nasal process include *Z. quadrifrons* [41], *D. zarudnyi* [43], *R. hatcherii* [44], and two amphisbaenian species [45]. In contrast, species with a longer posterior process of the nasal bone include *A. alba* [42] and *S. fossorium* [46]. The frontal bones interlock with each other in a tongue-and-groove articulation, which is particularly well developed towards the distal ends of the bones in three *Blanus* species. Three species of Amphisbaena [42,45], *R. hatcherii* [44], and *S. fossorium* [46] exhibit similarities to the three *Blanus* species examined, while in *Z. quadrifrons* [41] and *D. zarudnyi* [43], the sutures between the frontal bones are observed to be straight. *A. alba* [42], *A. arda*, and *A. vermicularis* [45] exhibit six finger-like projections on the parietal bones. The parietal notch is narrow and rounded in *A. arda* and *A. vermicularis* [45], while it is narrow and blunt in *A. alba* [42]. The premaxillary teeth consist of seven teeth in *Z. quadrifrons* [41], *A. alba* [42], species of *Blanus* [28], and *A. arda* and *A. vermicularis* [45], whereas the alveolar plates of *D. zarudnyi* [43], *S. fossorium* [46], and *R. hatcherii* [44] hold three teeth. The alveolar plate projects forward and develops into a rostral process. This condition is similar to that observed in *D. zarudnyi* [43], *Z. quadrifrons* [41], *R. hatcherii* [44], and species of *Blanus* [28]. However, this feature differs in other amphisbaenians, such as *A. alba* [42] and *A. arda* and *A. vermicularis* [45]. The facial process of the maxilla is wider than it is tall in *Z. quadrifrons* [41], *R. hatcherii* [44], species of *Blanus* [28], *A. alba* [42], *S. fossorium* [46], and *A. arda* and *A. vermicularis* [45]. However, *D. zarudnyi* [43] differs from other amphisbaenians in its longer body structure. On the dorsal part of the facial process of the maxilla, projections extend posterodorsally, with the first being the longest and pointed, while the others are shorter and rounder in the three *Blanus* species examined. The protrusions at the dorsal end of the facial process are single in *A. alba* [42], *A. arda* and *A. vermicularis* [45], *Z. quadrifrons* [41], *R. hatcherii* [44], and *S. fossorium* [46] and bifurcated in *D. zarudnyi* [43]. The base of the facial process of the maxilla is pierced in the anteroposterior direction by the superior alveolar canal located on the medial surface of the process. The number of labial foramina is two in *D. zarudnyi* [43] and *S. fossorium* [46], and the number is greater in *A. alba* [42], *R. hatcherii* [44], *B. cinereus*, and *B. vandellii* [28] and variable in *A. arda* and *A. vermicularis* [45]. The maxilla carries four teeth in *Blanus* species [28] and *A. arda* and *A. vermicularis* [45], three in *D. zarudnyi* [43] and *Z. quadrifrons* [41], six in *R. hatcherii* [44], and seven in *S. fossorium* [46]. The largest tooth in the maxillary bone varies among amphisbaenians. In three species of *Amphisbaena* [42,45], the first tooth is the largest, similar to species of *Blanus* [28]. In contrast, in *R. hatcherii* [44], *S. fossorium* [46], and *Z. quadrifrons* [41], the second tooth is the largest.

The fossil record of the genus *Blanus* has been the subject of several detailed morphological studies, particularly focusing on cranial and dental characteristics to clarify taxonomic relationships within the group. Delfino [30] reported the presence of eight teeth in the dentary, seven teeth in the premaxilla (with the middle tooth being the largest), and five teeth in the maxilla (where the first is very small and the second is the largest). In a later study, Čerňanskỳ and Venczel [31] could not provide detailed cranial and dentary character descriptions due to the limited and fragmentary nature of their specimen, which consisted of a small and insufficient sample. Bolet et al. [32] investigated the cranial and limited vertebral osteological characteristics of the fossil species *Blanus mendezi* sp. nov., focusing on the morphology of the premaxilla, quadrate, sutural structures, and dentary tooth arrangement, and revealed significant similarities with extant *Blanus* clades. Georgalis et al. [33] likewise noted a total of eight teeth on the dental bone. Most recently, Syromyatnikova et al. [34] provided a new *Blanus* fossil record, describing dental features—specifically, the presence of an enlarged second tooth and a small third dentary tooth—which support a close relationship with the *Blanus strauchi* complex (eastern clade).

The postcranial skeleton of *Blanus* also revealed interesting patterns, particularly in the vertebral column and pelvic structures. The number of presacral vertebrae in the three *Blanus* species examined showed notable differences, particularly in the thoracic and caudal vertebrae. The presacral vertebra counts are as follows: *Blanus aporus* < *B. alexandri* < *B. strauchi*. Variation in the number of presacral vertebrae among lizards reflects a complex interplay of phylogenetic history, ecological adaptation, and functional demands. Phylogenetic divergence contributes significantly to these differences, with specific vertebral structures serving as indicators of evolutionary relationships [47,48,49,50]. Functionally, the number of presacral vertebrae is closely linked to locomotor strategies: maneuverability typically requires a high degree of body flexibility, which is supported by a greater number of vertebrae relative to body length [51,52,53,54,55]. In contrast, a stiffer trunk, characterized by fewer vertebrae per unit body length, enhances maximum speed and acceleration capacity. Habitat type also plays a crucial role: lizards inhabiting open environments tend to have fewer presacral vertebrae, whereas those in structurally complex habitats possess more [55,56]. Overall, variation in presacral vertebrae provides valuable insights into the evolutionary trajectories and ecological specializations of lizards, highlighting the functional significance of axial morphology across diverse adaptive contexts.

Geometric morphometric analyses revealed distinct differences in dorsal and ventral cranial shapes among the three species. PCA effectively captured the variation in dorsal and ventral cranial morphology, with *B. alexandri* exhibiting more negative scores along PC1. Similarly, in the geometric morphometric analysis conducted by Sindoca et al. [2] based on the outer plate morphology of the dorsal skull, *B. alexandri* displayed a positive score on the PC2 axis, distinguishing it from *B. strauchi* and *B. aporus*. These shape differences provide additional evidence that the cranial morphology of *Blanus* species is subject to evolutionary pressures that influence their functional and ecological adaptations.

Phylogenetic analysis based on osteological data also yielded insights into the evolutionary relationships among *Blanus* species. Our results corroborate previous molecular findings, positioning *B. alexandri* as a distinct lineage from the closely related *B. aporus* and *B. strauchi*. While molecular studies [2] grouped *B. alexandri* and *B. aporus* as sister taxa, our osteological data suggest a more nuanced relationship, underscoring the importance of incorporating skeletal traits into phylogenetic frameworks, especially for taxa exhibiting subtle external morphological variation.

Ultimately, this study highlights the enduring relevance of osteological characters in taxonomy, particularly when integrated with molecular data. Furthermore, the observed variations in cranial and postcranial features could be indicative of ecological adaptations, with each species of *Blanus* evolving specific traits in response to their respective environments. In conclusion, this research emphasizes the importance of integrating osteological and geometric morphometric approaches to study the evolutionary dynamics of *Blanus* and related taxa. Future studies incorporating a broader sampling of amphisbaenian taxa and more detailed morphological data will further clarify evolutionary relationships and help elucidate the adaptive strategies underlying skeletal variation in these cryptic reptiles.

## Figures and Tables

**Table 1:** Description of specimens.

Species	Region (City of Türkiye)	Number of Sample
*Blanus alexandri*	Adıyaman	3
	Mardin	2
	Urfa	3
*Blanus aporus*	Antalya	2
	Adana	2
	Mersin	2
	Hatay	2
*Blanus strauchi*	Marmaris (Muğla)	3
	Fethiye (Muğla)	3
	Muğla	2

**Table 2:** The average number of vertebrae in three *Blanus* species.

Species	Cervical Vertebrae	Thoracic Vertebra	Lumbar Vertebrae	Caudal Vertebrae
*B. aporus*	2 (atlas and axis)	98	2	16
*B. alexandri*	2 (atlas and axis)	100	2	19
*B. strauchi*	2 (atlas and axis)	105	2	19

## Data Availability

The original contributions presented in this study are included in the article.

## Appendix A

**Table A1:** Summary of the osteological data available for amphisbaenians.

Species	Osteological Data	Source
*Amphisbaenian* sp.	Postcranial osteology	[26]
*Amphisbaenian* sp.	Postcranial osteology	[26]
*Amphisbaenian* sp.	Postcranial osteology	[27]
*Slavoia darevskii* (*extinct* sp.)	Postcranial osteology	[29]
*Zygaspis quadrifrons*	Postcranial osteology	[57]
*Blanus* sp.	Cranial osteology	[28]
*Blanus mendezi* sp. *Nov.* (*extict* sp.)	Cranial osteology	[32]
*Amphisbaenian* sp.	Cranial osteology	[36]
*Zygaspis quadrifrons*	Cranial osteology	[41]
*Amphisbaenia alba*	Cranial osteology	[42]
*Diplometopon zarudnyi*	Cranial osteology	[43]
*Rhineura hatcherii* (*extinct* sp.)	Cranial osteology	[44]
*Spathorhynchus fossorium* (*extinct* sp.)	Cranial osteology	[46]
*Amphisbaenian* sp.	Cranial osteology	[58]
*Diplometalon zarudnyi*	Cranial osteology	[59]
*Genus Zygaspis*	Cranial osteology	[60]
*Diplometopon zarudnyi*	Cranial osteology	[61]
*Amphisbaenian* sp.	Cranial osteology	[62]

## Appendix B

The list for observed variation in key osteological traits, offering a detailed comparison of their presence, form, and structure.

1. Premaxilla: the number of teeth: (0) 3, (1) 7.
2. Premaxilla: rostrol process: (0) absent, (1) present.
3. Premaxilla: ascending nasal process: (0) tapers posteriorly with a slight projection, (1) tapers posteriorly with a slight depression, (2) narrows towards the truncated or pointed dorsal end, (3) tapers with an arrow shaped*, (4) narrow and elongated end*, (5) appears to be short and peg-like, (6) tapers posteriorly with rounded end.
4. Premaxilla: ascending nasal process contacts to: (0) anteromedial process of the frontal, (1) nasal.
5. Maxilla: The number of teeth: (0) 3, (1) 4, (2) 5, (3) 6, (4) 7.
6. Maxilla: the larger teeth: (0) first teeth, (1) second teeth.
7. Maxilla: facial process: (0) wider, (1) taller.
8. Maxilla: the number of the projection from the dorsal end of the facial process: (0) 1, (1) 2, (2) 3.
9. Maxilla: the number of labial foramen: (0) 1, (1) 2, (2) more than two, (3) variable, (4) more than two and each side variable.
10. Nasal: the posterior process of the nasal: (0) the posterior process of the nasal bone is shorter than the length of the distal portion of the ascending nasal process of the premaxilla, (1) the posterior process of the nasal bone is approximately equal in length to the distal portion of the ascending nasal process of the premaxilla. (2) the posterior process of the nasal bone is longer than the length of the distal portion of the ascending nasal process of the premaxilla.
11. Frontal: suture between the frontals: (0) straight articulation, (1) tongue-and-groove articulation.
12. Frontal: number of maximum finger-like projections on the posterior margin of each frontal bone: (0) 3, (1) 4, (2) 5, (3) 6, (4) 7.
13. Parietal: number of maximum finger-like projections on the anterior margin of the parietal bone: (0) 5, (1) 6, (2) 7, (3) 8, (4) 9, (5) 10, (6) 11.
14. Parietal: parietal notch: (0) narrow, (1) wide.
15. Parietal: the lateralmost finger-like projection touches the prefrontal: (0) yes, (1) no, (2) both, (3) approach but not contact.
16. Parietal: parietal (pineal) foramen: (0) present, (1) absent.
17. Parietal: sagittal crest; (0) distinct, (1) indistinct, (3) absent.
18. Parietal: otic-occipital lappet; (0) wide, (1) narrow.
19. Parietal: the length of the finger-like projection; (0) relatively equal, (1) not equal.
20. Prefrontal: (0) present, (1) absent.
21. Squamosal: the length of the bone: (0) present, (1) absent, (2) fused with the otic-occipital complex.
22. Quadrate: the projection of the posteromedial part of the pillar: (0) distinct, (1) thin, (2) poor development.
23. Quadrate: quadrate foramen; (0) present and one, (1) absent, (2) present and more than one.
24. Epipterygoid: (0) present, (1) absent, (2) both.
25. Vomer: rostral process: (0) bifurcated, (1) long and slender, (2) wide and triangular-shaped posteriorly.
26. Vomer: posterior process: (0) long and narrow with an acute end, (1) long and slender.
27. Vomer: lateral wings: (0) slender and anteriorly directed, (1) posteriorly directed and then tapers, (2) posteriorly directed and widens, (3) widens laterally at approximately its midpoint, (4) widens and anteriorly directed, (5) slender and posteriorly directed.
28. Palatine: the laminar expansion of the lateral margin of the bone: (0) long, (1) short, (2) poor development, (3) absent.
29. Palatine: anterior processes: (0) the length of the vomerine process > the maxillar process, (1) the length of the vomerine process < the maxillar process, (2) the length of the vomerine process = the maxillar process, (3) absent.
30. Palatine: pterygoid process: (0) laterally oriented, long, and pointed, (1) flat and ends in a pointed tip, (2) projects posteriorly, (3) posteriorly directed and further tapers.
31. Palatine: palatine foramen: (0) present, (1) absent.
32. Pterygoid recess: (0) straight, (1) wide angle, (2) narrow angle, (3) absent.
33. Pterygoid: teeth: (1) present, (2) absent.
34. Dentary: the longest tooth of the dentary: (0) first, (1) second, (2) third, (3) first and forth.
35. Dentary: the number of ventral posterior process of the dentary: (0) 0, (1) 1, (2) 2, (3) 3.
36. Dentary: the first foramen of the dentary: (0) the level of the 2–3 teeth, (1) the level of the 1–2 teeth, (2) the level of the 1 teeth, (3) on the 2th teeth, (4) mental foramina are variable on the left and right sides.
37. Dentary: The second foramen of the dentary: (0) the level of the 3–4 teeth, (1) the level of the 2–3 teeth, (2) on the 2nd teeth, (3) on the 4th teeth, (4) mental foramina are variable on the left and right sides.
38. Dentary: The third foramen of the dentary: (0) the level of the 6–7 teeth, (1) the level of the 5–6 teeth, (2) the level of 3–4 teeth, (3) the level of 4–5 teeth, (4) on the 5th teeth, (5) mental foramina are variable on the left and right sides.
39. Dentary: the number of teeth: (0) 6, (1) 7, (2) 8, (3) 7 or 8.
40. Presacral vertebra: the average number of the presacral vertebrae: (0) 118, (1) 123, (2) 128.

## Appendix C

Data matrix of *Blanus* group and outgroup taxa. Symbol “?”: not applicable. See Appendix B for descriptions of osteological characters.

**Species**	**Data Matrix**	**Reference**	**Citations**
*Blanus alexandri*	1100100310135111100001020001001112330031	Present Study	
*Blanus aporus*	1110100310125111100000020001001112315030	Present Study	
*Blanus strauchi*	1110100240136131100010220001001112310032	Present Study	
*Blanus cineraus*	112010012011301100000100000100111233003?	Villa et al., 2019	[28]
*Blanus tingitanus*	112010012012311100001101000100111231002?	Villa et al., 2019	[28]
*Blanus mettatali*	11201001301131?100000200000100111231003?	Villa et al., 2019	[28]
*Blanus vandelli*	112010012011301100000200000100111231003?	Villa et al., 2019	[28]
*Amphisbaena alba*	10402000221120210100?20?002???111230302?	Montero & Gans, 1999	[42]
*Amphisbaena arda*	10401000311120210000??0?102?021?1224451?	Paiva et al., 2024	[45]
*Amphisbaena vermicularis*	10401000311100210000??0?102?021?1224451?	Paiva et al., 2024	[45]
*Zygaspis quadrifrons*	11160100210430111000?10?005300011222233?	Bell et al., 2023	[41]
*Diplometopon zarudyni*	01300011210151110011001?004302031003110?	Maisano et al., 2006	[43]
*Rhineura hatcherii*	01513100211??0010110221?003302121331141?	Kearney et al., 2005	[44]
*Spathorhychus fossorium*	0?614100221???010??0211?201?230?1332222?	Müller et al., 2016	[46]
